# Evaluation of a Revised Home Medication Experience Questionnaire (HOME-Qv2)

**DOI:** 10.3390/pharmacy8030169

**Published:** 2020-09-11

**Authors:** Matthew J. Witry, Olajide O. Fadare, Kassi Pham

**Affiliations:** College of Pharmacy, The University of Iowa, Iowa City, IA 52245, USA; olajide-fadare@uiowa.edu (O.O.F.); kassi-pham@uiowa.edu (K.P.)

**Keywords:** medication experience, patient, pharmacist, adherence, questionnaire, medication beliefs

## Abstract

The Home Medication Experience Questionnaire (HOME-Qv2) was developed to identify patient medication experience issues for pharmacist intervention. The study objectives were to (1) evaluate patient responses to the HOME-Qv2 medication experience questionnaire and (2) describe recommendations made by pharmacists in response to the identified medication experience issues and adoption of recommendations. The study sample was comprised of older adults, 55 years and above, who have one or more chronic illnesses for which they routinely take 4+ prescription medications. The HOME-Qv2 was administered to patients and a pharmacist made recommendations based on the responses. At 3 months, the research team followed up with participants via telephone, during which the HOME-Qv2 was again administered and participants shared their adoption of recommendations. Twenty-four patients completed the questionnaire, and twenty-one were available for follow-up. At 3 months, there was a significant decrease in patient self-reported HOME-Qv2 medication experience issues. There were 31 interventions/recommendations provided by the pharmacists, and 64.5% reported adopted. The HOME-Qv2 appears to facilitate patient disclosure of medication experience issues and informed targeted pharmacist recommendations.

## 1. Introduction

Medications, including over the counter (OTC) [1], dietary supplements [2], and prescription recommended for acute and chronic conditions are the most common treatment option employed in the provision of healthcare [3]. As medication advances have extended lives and reduced the mortality rate for many diseases, the accompanying prevalence of multimorbidity associated with increasing age creates a scenario in which older adults require complex medication regimens (polypharmacy) to manage their health [4,5,6]. The high rates of multimorbidity among older adults in the U.S. [7], combined with care practices that promote overprescribing rather than prioritize medication monitoring [8] have made polypharmacy a defining feature of the patient medication experience [4,9]. Polypharmacy is not without risks and has been associated with increased healthcare costs from drug therapy problems related to sub-optimal medication use [10], adverse events and lower quality of life [11].

Historically, the focus of pharmacists has been on identifying and resolving medication-related problems by using their knowledge of pharmacotherapy [12]. This approach prioritizes verifying that every medication a patient takes is appropriate and effective for the indication, safe-given patient characteristics, and that the patient has access to the medication [13]. While this is an important pursuit, and patients will always need the right medication at the right dose at the right time, an emerging focus for the healthcare system is to move from discrete service offerings, such as medication regimen reviews, to following patients holistically over time in a continuous medication monitoring orientation [14]. This approach gives more consideration to patients as individuals who have unique preferences and lived experiences with their medications that shape their adherence to treatment and, ultimately, their outcomes [15].

Shoemaker et al. defined patient medication experience as “… an individual’s subjective experience of taking a medication in [their] daily life. It begins as an encounter with a chronic medication. It is an encounter that is given meaning before it happens and is often a reaction to the symbol that medication holds. The experience may include positive or negative bodily effects. The unremitting nature of a chronic medication often causes an individual to question the need for the medication. Subsequently, the individual may exert control by altering the way he takes the medication and often in part because of the gained expertise with the medication in his own body [16].” These lived medication experiences have been documented across a variety of conditions, backgrounds, and settings [17].

**Figure 1:**
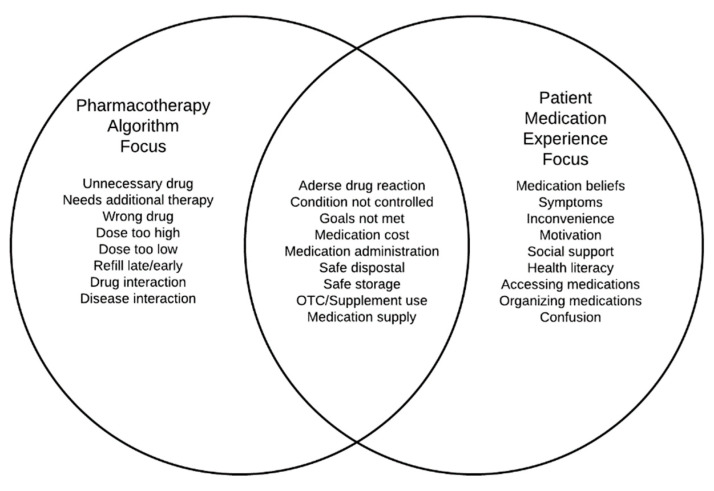
Contrasting the pharmacotherapy algorithm focus, a patient medication experience focus, and areas where the two foci may overlap.

Information about the patient’s medication experience cannot be revealed through either a pharmacotherapy-focused algorithm or a software suite which are better suited for identifying issues, such as out of range dosing and medication interactions (Figure 1). Further, someone’s medication experience is not necessarily included when evaluating for adherence to guideline-recommended treatments. Similarly, an alert indicating an early or late refill does not tell the pharmacist the reason for apparent non-adherence. Rather, it is up to the skilled and inquisitive pharmacist to uncover medication experience issues, idiosyncrasies, beliefs, and practices that shape how they use their medications when they get home [15]. There also are important ways a pharmacotherapy algorithm focus, or as some have labeled it the “pharmaceutical gaze [18,19]”, and the medication experience focus can overlap (Figure 1). For example, a patient volunteering a symptom of dizziness upon standing could inform a pharmacist’s examination of their profile for an antihypertensive medication that commonly causes orthostatic hypotension.

However, many patients do not actively engage with pharmacists and volunteer information without prompting [20]. Additionally, even when asked, patients may provide socially desirable responses or avoid engaging with a pharmacist that they may not be familiar with [21], or comfortable sharing their beliefs and experiences with [22]. As pharmacies are busy and patients have a range of concerns, the Home Medication Experience Questionnaire (HOME-Q) was developed and piloted to provide patients with an opportunity to share their medication experiences for the pharmacist to use in the medication monitoring process [23,24]. The HOME-Q is not intended as a risk assessment tool, but rather to identify medication experience issues that may warrant a discussion with a pharmacist who may be able to offer education or engage in other problem-solving approaches.

In this study, a revised version, the HOME-Qv2 was developed based on several findings from the initial HOME-Q evaluation and trialed in a new sample of patients. The study objectives were to (1) evaluate patient responses to the HOME-Qv2 medication experience questionnaire (Supplementary Materials) and (2) describe recommendations provided by pharmacists in response to the identified medication experience issues and patients’ adoption of these recommendations. 

## 2. Materials and Methods

Approval was obtained for this study from the institutional review board (IRB) of the university (#201710776). Two independently owned community pharmacies were recruited into the study. The questionnaire was administered to a purposeful sample of pharmacy patrons aged 55 years or older who have one or more long-term illnesses (e.g., diabetes, hypertension) for which they take 4 or more chronic prescription medications. The rationale for this sample selection is to identify patients who are at higher risk for medication-related problems due to their complex treatment regimens.

### 2.1. Revisions to the Initial Version of the HOME-Q

A revised version of the HOME-Q [23] was used in the present study after making 3 modifications. The first change was to drop or modify some items that were not associated with meaningful pharmacist actions. The second change was to re-orient and positively phrase half of the items to reflect best practices. The rationale was to give participants another way to disclose information about their medication experience. The third change was to introduce a middle “sometimes” response. This change was in response to some participants completing the original HOME-Q marked in-between the binary yes/no options which we inferred as their wanting to express a partial, or sometimes response. We also thought patients may be more comfortable disclosing a possibly socially undesirable response if given a middle option.

### 2.2. Study Process

Four pharmacists, who were either residency trained or current residents at the time, partnered with the research team to obtain informed consent and deliver the HOME-Qv2 interventions. The participating pharmacists were trained on the study protocol and in the use of several communication techniques to facilitate an interactive environment where patients could comfortably disclose medication experience issues or beliefs about medicines.

Study pharmacists used purposeful sampling to recruit patients during routine patient–pharmacist interactions that occurred while patients picked up their medicines at the pharmacy by asking them if they would be interested in taking a survey about their medication use experience in the home. Those who indicated interest received further information from the pharmacist about the study. After obtaining written consent, the pharmacists provided the patients with a study packet that contained a paper version of the HOME-Qv2. Patients had the option to complete the questionnaire in the pharmacy or take it home to complete. Those who took the questionnaire home received a postage-paid return envelope for them to mail the completed form back to the pharmacy.

At a pre-arranged time, the pharmacists and patients met, either in-person or over the phone, to discuss patient responses to the HOME-Qv2, during which pharmacists made recommendations to address emergent medication experience issues. Patients received USD 10 compensation for taking the survey and meeting with the pharmacist, and another USD 10 for being part of the 3-month follow up call. The participating pharmacies were compensated USD 75 per patient recruited, consented, and met with for the intervention. One pharmacy used their own electronic records system to document pharmacist–patient encounters while the second pharmacy used a paper form provided by the research team.

At 3 months, a follow-up telephone call was made by a member of the research team to study participants to re-administer the HOME-Qv2, and to assess if patients adopted and maintained the pharmacist’s recommendations. The research team considered 3 months between the first and second administration of the questionnaire to be enough time for patients to adopt recommendations while minimizing the risk of patients forgetting about their previous interaction with the pharmacist. Participants were asked to give feedback on the survey or their encounter with the pharmacist if they had any. The calls were not audio recorded, rather, handwritten notes were documented by the research assistant.

### 2.3. Analysis

Frequencies were calculated for each risk-factor item, and each best-practice item on the HOME-Qv2 at baseline (time 1) and at three months (time 2). Continuous variables were assessed for normality by examining histograms, skew, kurtosis, and by using the Shapiro–Wilk test. Medians and interquartile range (IQR) were calculated for continuous variables with non-normal distribution. HOME-Qv2 totals at baseline and at 3 months were calculated, and the differences were assessed using the Wilcoxon signed rank test with an a priori 0.05 level of significance.

The HOME-Qv2 items are conceptualized as prompts intended to identify patients’ medication experience issues, concerns, and practices. A patient’s affirmative response to a risk factor phrased item (1–10) was expected to elicit a discussion, and for some, an intervention or recommendation from the pharmacist. For the purposes of the study, a “Yes” answer was assigned 1 point and “Sometimes” was assigned 0.5 points. A patient’s contradictory response to a best-practices phrased item (11–20) is similarly expected to elicit a discussion, and for some, an intervention or recommendation. For the purposes of the study, a “No” answer was assigned 1 point and “Somewhat” was assigned 0.5 points. The HOME-Qv2 totals (“Yes” plus “Sometimes” for risk factor subscale, and “No” plus “Somewhat” for the best-practices subscale), and medians for each subscale are calculated at baseline and after 3 months. The percentage contribution of the “Somewhat/Sometimes” response option to the identification of patient medication experience issues is determined. Two authors collaborated to count and descriptively code the pharmacist interventions into categories using a similar process used to evaluate the initial version of the HOME-Q.

## 3. Results

Twenty-four participants were enrolled and completed an initial encounter, but three patients could not be reached to complete a follow-up encounter at the 3-month call and were excluded from the final analysis. Twenty-one participants who had complete data for both rounds of the survey were included in this analysis. The average age of the participants was 70 (SD = 9.55), and 12 (57.1%) were men. The median number of oral medications was 6 (IQR = 3).

At baseline, there were a total of 101 medication experience issues (45 yes/no at 1 pt ea and 56 sometimes at 0.5 pt ea) reported in response to the 20 HOME-Qv2 items (median = 3.5, IQR = 3.0). At follow up, there were 60 medication experience issues (35 yes/no at 1 pt ea and 25 sometimes at 0.5 pt ea) reported (median = 1.5, IQR = 1.5) and the difference was significant (Table 1).

The “sometimes” response option accounted for 53.4% (31/58) of the positive baseline responses on the risk-factor subscale and accounted for 36.5% (15.5/42.5) of the points. The per-respondent median for the risk-factor subscale was 3.0 (IQR = 2.375) at baseline and 2.0 (IQR = 0.75) at 3 months. On the best-practice subscale, (where a positive value corresponds to not using the best practice), the “somewhat” response option accounted for 58.1% (25/43) of the positive baseline responses and accounted for 41.0% (12.5/30.5) of the points. The per-respondent best-practice subscale median was 2.5 (IQR = 2.625) at baseline and 1.0 (IQR = 1.0) at 3 months.

Respondents reported a variety of risk-factor items on the baseline HOME-Qv2 (Table 1), which include yes or somewhat responses, such as if lowering the cost of their medication would be helpful (19/21), wondering if there are medicines they should not be taking anymore (9/21), and wondering if their medicines were doing more harm than good (7/21). For the best-practice subscale, respondents disagreed or reported somewhat that the cost of their medicines fits their budget (10/21) and that they use a medication organizer (6/21). At 3 months, reductions were observed for items such as the number expressing concern about the costs and safety of their medications and being unsure about the best time to take medicines.

There were 31 coded recommendations across seven categories (Table 2). These categories are safety and proper use of medications (e.g., “take famotidine 30 min before breakfast and evening meals”) (n = 5), cost cutting (e.g., “discuss with PharmD to evaluate cheaper alternative to medicines”) (n = 8), medication review and referral to a physician (e.g., “PharmD educates patient on need to take lisinopril for BP management,” and “follow up with doctor on pain in shoulder that may be associated with Crestor”) (n = 7), organizing medications (e.g., “compliance packaging options available as alternatives for med boxes”) (n = 5), enlisting family support (e.g., “suggested family members remind him to bring his pills along when going out”) (n = 2), proper medication disposal (e.g., “dispose meds (not needed) through pharmacy”) (n = 2), diet and exercise (e.g., “informed patient about diet and exercise as a way to decrease insulin dose”) (n = 2).

All but two of the categories of recommendations had at least 50% acceptance. Recommendations to address cost concerns had 25% acceptance (2/8), and recommendations on organizing and planning medication use had 40% acceptance (2/5). Recommendations about proper medication use, proper disposal of medications no longer needed, and about diet and exercise reported 100% acceptance. The number of interventions per patient ranged from 0 to 4, and 20 of the interventions (64.5%) appeared to be adopted and/or maintained at the 3-month call.

## 4. Discussions

The HOME-Q was developed to facilitate patient disclosure of their home medication experience issues and practices, such that responses could lead to discussions and problem-solving with the pharmacist and prescribers when necessary [23]. The revised version, HOME-Qv2, used in this study included items designed to uncover home medication experience issues, concerns, and best practices which may not yet have been adopted to expand the scope of information available to the pharmacist about their patient’s medication experience for providing guidance, education, and recommendations. For the study sample, there was a significant decrease in HOME-Qv2 scores over the 3-month follow-up period (*p* = 0.008) which suggests some of the pharmacist recommendations may have resolved issues and concerns and promoted the adoption of medication experience best practices. These data also suggest the addition of a middle option and phrasing half of the items as best practices were beneficial changes.

Study data show a decrease in patient-reported concern about medication safety (“I wonder if my medicines are doing more harm than good”) and belief that they might be taking medicines that they do not need, at 3 months. This decrease suggests these concerns may have been addressed by the pharmacists’ counsel about the safety and proper use of medicines. Example actions in this study included educating a hypertensive patient about the unique value of lisinopril or advising on the prevention of adverse events for medications such as celecoxib. Studies suggest medication adherence largely relies on patients making intentional decisions [25,26].

Concerns related to the cost of medicines were the most self-reported medication experience issue, but related recommendations had the lowest acceptance rate. While how much medications cost is usually beyond the control of patients, pharmacists are sometimes, but not always, able to improve the medication experience by helping make medicines more affordable. For instance, one of the pharmacist’s interventions to address cost that was accepted was to evaluate cheaper alternatives for the patient, which is considered a viable approach to cost-saving [27,28].

For this study sample, when asked about their interest in having their medicines packaged, and the use of medication planning tools, some respondents indicated interest, but packaging solutions recommended by the pharmacists were generally not accepted. Patients may not be favorably disposed to adopting medication packaging solutions especially if there is added cost [29,30]. This is despite some evidence that packaging solutions can help some patients better adhere to their regimens [31,32].

While adherence packaging was not employed as a result of any of the encounters, patients did report adopting pharmacist recommendations to enlist the help of family members in developing and maintaining a convenient routine for using their medications as prescribed, with fewer reports of not taking medicines when there is a change in schedule recorded at the 3-month follow-up encounter. Such a recommendation could be particularly useful for older adults who might be experiencing some cognitive and physical limitations that add to the complexity of managing their medications daily [33].

On the first version of the HOME-Q, patients did not seem to respond positively to an item that reported they keep old prescription medicines just in case. The item was revised because it was not associated with meaningful pharmacist actions. The HOME-Qv2 introduced an item about the disposal of medicines which seemed to resonate better as a best practice. An increase in awareness of how to properly dispose of medicines may reduce the potential for accidental poisoning and drug misuse by recommending a pathway to remove excess or unused over-the-counter and prescription medicines from the home [34].

The pharmacist interventions provided in the study were personalized to the HOME-Qv2 responses of each patient who participated in the study. This case-specific approach to addressing medication experience issues may help further develop the relationship between the patient and the pharmacist [35]. Anecdotally, positive comments about the pharmacist were expressed to the research team during the 3-month telephone follow-up call.

The revised version, HOME-Qv2, used in this study added middle response options “sometimes” and “somewhat” to the questionnaire. At baseline, when the patient and pharmacist were engaging in their HOME-Q intervention, these middle options were chosen more often than the full yes/no response for the two subscales (53.4% and 58.1% of the total responses, respectively). This suggests the middle response option gave patients a way to express themselves on issues where they would be conflicted to make a binary yes/no response. Since the goal of the HOME-Qv2 is to facilitate a discussion about medication experience, the sometimes option may have given the patient and pharmacist more medication experience topics to discuss. In addition, the HOME-Qv2 introduced items aimed at initiating discussions about best practices that could contribute to a better medication experience for the patient. Overall, the dual focus of the questionnaire may have enabled the pharmacist to explore the links between best practices, risk factors, and medication experience to provide interventions that address a variety of patient needs and perspectives.

We posit that the revisions made to the HOME-Qv2 improved the value of the questionnaire. This is supported by the HOME-Qv2 yielding a higher proportion of adopted recommendations and a significant decrease in patient self-reported medication experience issues at the 3-month call, compared to no change with the initial version of the HOME-Q. However, there is a need for future work to further evaluate and validate this patient engagement tool.

As the aim of the HOME-Qv2 is to help providers characterize and assess the home medication experience of patients, further studies are needed to validate if the HOME-Qv2 captures medication experience issues that would otherwise have been missed without the questionnaire, and to evaluate the interventions that derive from using the HOME-Qv2 for longer-term impact. Validation work also is needed to determine what point value should be assigned to the sometimes response or if all positive responses should be treated the same. Additionally, assessment of how the HOME-Qv2 impacts patient–provider discussions about patient experience with using and managing medications in the home, and whether using the HOME-Qv2 enables pharmacist–patient communication that facilitates trust-building and disclosure would provide valuable information about the questionnaire’s value [12,16,36,37], including how the questionnaire may integrate within existing medication workups such as comprehensive medication reviews and chronic care management. Also, more research is needed to enhance the salience of the HOME-Qv2 as a medication experience assessment tool by incorporating medication experience issues that may be relevant to different demographic and socioeconomic groups, as well as chronic conditions.

This study has several limitations. The small sample size and the limited diversity of patients and pharmacists who participated in the study diminish external validity. Additional trials with more diverse patient populations are needed to increase generalizability. Selection bias due to non-random sampling may limit internal validity. The study was not designed to differentiate between modes of administration (telephone and in-person) and patients may have responded differently when they knew their questionnaire was going to be viewed by the pharmacist as part of the visit and the 3-month call which was conducted by a member of the research team. Further, the questionnaire has not yet been tested for test–retest reliability.

## 5. Conclusions

The HOME-Qv2 helped pharmacists identify medication experience issues and their frequency of occurrence in the study sample prompting discussions and recommendations from the pharmacists. A high proportion of the recommendations were adopted and there was a significant decrease in the reported medication experience issues after 3 months.

## Figures and Tables

**Table 1:** Description of Home Medication Experience Questionnaire (Home-Qv2) initial and follow-up responses (n = 21).

Item No.	HOME-Qv2 Items	Initial (T1)	Follow-Up (T2)	Change (T1–T2)
	Risk-Factor Subscale	Yes/Sometimes ^2^	Yes/Sometimes ^2^	
5	Lowering my medicine costs would be helpful	18	15	3
8	I wonder if there are medicines that I don’t need to be taking anymore	6	2.5	4.5
7	I wonder if my medicines are doing more harm than good	4	1.5	2.5
1	I am unsure about the best time to take my medicines	3	2	1
2	I am interested in having my medicines packaged for me	3	0	3
10	I feel unsteady when standing or walking	3.5	5.5	−2
9	I use more than one pharmacy to get my everyday medicines	1.5	0	1.5
6	I miss doses of my everyday medicines	1.5	2	−0.5
3	I get confused about if I took a medicine or not	1	2	−1
4	I have days where I have run out of my medicines	1	2	−1
	Best-Practice Subscale	No/Somewhat ^2^	No/Somewhat ^2^	
15	The cost of my medicines fits my budget	7	2	5
12	I use an organizer or pillbox for my medicines	5.5	4	1.5
13	I take my medicines when there is a change to my schedule	4.5	2	2.5
16	My current medicines do not cause me problems	4	2	2
17	I feel good about the medicines I take	2.5	1	1.5
19	I know how to dispose of medicines that I don’t need	2.5	1	1.5
20	My doctor and pharmacist know the vitamins and supplements I take	2.5	0.5	2
18	I know what to do if I miss a dose of my medicine	1.5	1	0.5
11	I have an up-to-date list of my medicines	0.5	1	−0.5
14	Taking my medicines has become part of my daily routine	0.0	0.5	−0.5
	HOME-Qv2 Points Total Sample	73	47.5	*p* = 0.008
	Median Score (IQR ^1^)	3.5 (3.0)	1.5 (1.5)	

^1^ IQR = interquartile range. ^2^ Sometimes/somewhat = 0.5 point.

**Table 2:** Pharmacist interventions and patient-reported recommendation adoption.

Pharmacist Recommendation/ Action	N	Adopted/Maintained N (%)
Safety and proper use of medications	5	5 (100)
Cost management	8	2 (25.0)
Medication review/Refer to physician	7	5 (71.4)
Organizing medications (pillbox, reminders)	5	2 (40)
Enlisting family support	2	2 (100)
Proper medication disposal	2	2 (100)
Diet and exercise	2	2 (100)
Total	31	20 (64.5)

## Supplementary Materials

The following supplementary materials are available online at https://www.mdpi.com/2226-4787/8/3/169/s1, Supplementary: Questionnaire about how you take your medicines at home.
