# Supplementary material for: Evaluation of a Revised Home Medication Experience Questionnaire (HOME-Qv2)
Source: Pharmacy (Basel). 2020 Sep 11;8(3):169. doi: 10.3390/pharmacy8030169 (PMC7557765; doi:10.3390/pharmacy8030169)
Supplement: Supplementary file 1 [file pharmacy-08-00169-s001.pdf]

Questionnaire about how you take your medicines at home

Please mark yes, sometimes, or no for these statements. There are no right or wrong answers. Your feedback will help your pharmacist better understand you and your medicines.

	**Do you agree with the following statements?**	**Yes**	**Some-times**	**No**
1	I am unsure about the **best time** to take my medicines			
2	I am interested in having my **medicines packaged** for me			
3	I get confused about if I took a medicine or not			
4	I have days where I have **run out** of my medicines			
5	Lowering my **medicine costs** would be helpful			
6	I miss doses of my **everyday medicines**			
7	I wonder if my medicines are doing **more harm than good**			
8	I wonder if there are medicines that **I don't need** to be taking anymore			
9	I use **more than one pharmacy** to get my everyday medicines			
10	I feel **unsteady** when standing or walking			

These next questions are about things people do to manage their medicines safely at home. And their feelings about their medicines. Which ones are true for you?

	**Do you agree with the following statements?**	**Yes**	**Some-what**	**No**
11	I have an **up-to-date list** of my medicines			
12	I use an **organizer or pillbox** for my medicines			
13	I take my medicines when there is a change to my schedule			
14	Taking my medicines has become part of my daily **routine**			
15	The cost of my medicines fits my **budget**			
16	My current medicines do not cause me **problems**			
17	I feel good about the **medicines I take**			
18	I know what to do if **I miss a dose** of my medicine			
19	I know how to **dispose** of medicines that I don't need			
20	My doctor and pharmacist know the **vitamins and supplements** I take			
